# The Mystery of Low Phosphate: Marijuana is the Smoking Gun

**DOI:** 10.7759/cureus.7392

**Published:** 2020-03-24

**Authors:** Talha Ahmed, Safwan Muhammad, Ayesha Safdar, Amna Shaukat

**Affiliations:** 1 Internal Medicine, University of Maryland Medical Center, Baltimore, USA; 2 Internal Medicine, University of Maryland Medical Center Midtown Campus, Baltimore, USA; 3 Internal Medicine, Army Medical College, Rawalpindi, PAK; 4 Medicine, Services Institute of Medical Sciences, Lahore, PAK

**Keywords:** hypophosphatemia, marijuana, trans-cellular shifts, cannabis

## Abstract

Hypophosphatemia is a rarely reported side-effect of cannabis use. The potential mechanisms of hypophosphatemia include enteric malabsorption/loss, excessive urinary excretion, or rapid trans-cellular shifts. Severe hypophosphatemia from daily marijuana use is a rare side-effect. A trans-cellular shift is the most likely proposed mechanism. Although it tends to self-correct fairly rapidly, close observation for the dreaded consequences related to hypophosphatemia is required. Both the users and providers must be aware of this rare association of hypophosphatemia with daily marijuana use.

## Introduction

Cannabis is the most prevalent illicit drug used in the United States and is legal in some states. Though publicly accepted as an innocuous substance, there are potential adverse effects, which include impaired concentration, loss of memory, altered mood, and hyperemesis syndrome [1-2]. We report a rare association of severe hypophosphatemia in a cannabis user. Our patient initially presented with cannabis hyperemesis syndrome. With the cessation of marijuana use, hypophosphatemia corrected fairly rapidly. There have been case reports and case series showing the association of daily cannabis use with hypophosphatemia. Without cessation of cannabis use, the effects of severe prolonged hypophosphatemia are not known and hence both users and providers must be aware of this rare but potentially fatal adverse effect [3].

## Case presentation

**Figure 1:**
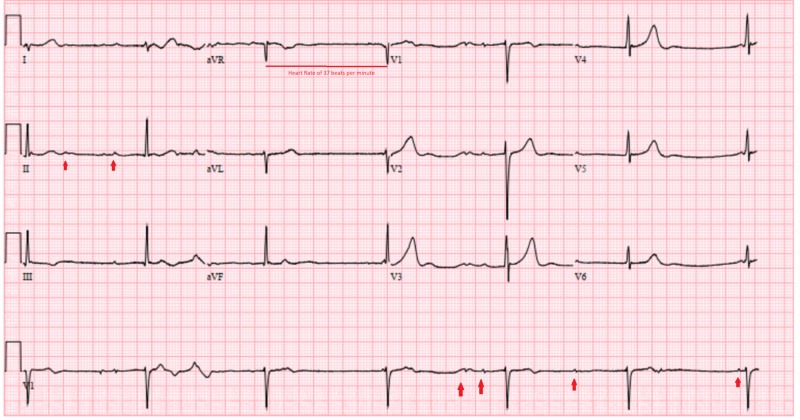
Electrocardiogram with a junctional rhythm at a rate of 37 beats per minute and atrioventricular dissociation

A 25-year-old male with a history significant for marijuana use, presented with abdominal pain, nausea, and occasional vomiting for two days. He was somnolent, though responsive, at the time of admission, with a heart rate of 36 beats per minute (bmp), blood pressure of 120/75 mmHg, and afebrile with oxygen saturation of 95 % on room air. The physical examination revealed bilateral conjunctival injection but was otherwise normal. Electrocardiogram (EKG) revealed a junctional rhythm with a heart rate of 37 bmp and atrioventricular (A-V) dissociation (Figure 1).

**Figure 2:**
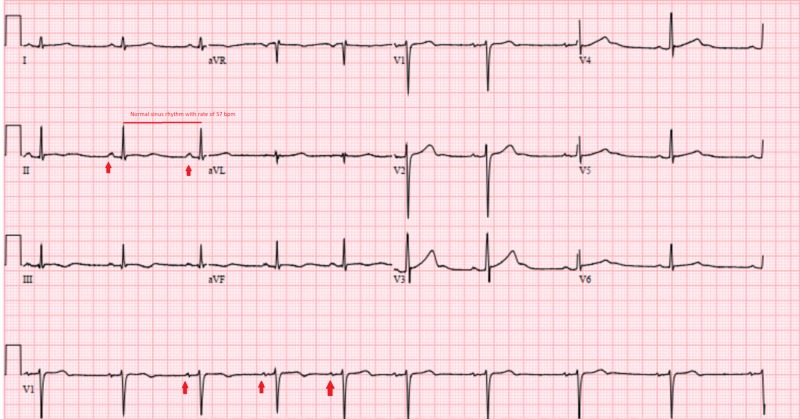
Electrocardiogram after correction of serum phosphate levels showed normal sinus rhythm with a heart rate of 57 bmp

The serum metabolic panel was remarkable for undetectable phosphorus level (<0.5 mg/dL) (normal range 2.5 - 4.5 mg/dl) but was otherwise unremarkable with a normal renal function. The urine toxicology screen was positive for cannabis and opiates. Urine analysis was unremarkable. The patient was commenced on intravenous potassium phosphate 60 mmol bolus. A few hours later, repeat laboratory testing showed rapid correction of his phosphorus level to 4.3 mg/dL, which was out of proportion to the amount of repletion that he received. An EKG done on the following day showed normal sinus rhythm with a heart rate of 57 bmp (Figure 2).

A careful review of this patient’s previous admission for cannabis hyperemesis syndrome showed similar dynamics of metabolic profile, in which his undetectable phosphorous levels corrected very rapidly to normal within a few hours.

## Discussion

Hypophosphatemia is a rarely reported side-effect of cannabis use. The potential mechanisms of hypophosphatemia include enteric malabsorption/loss, excessive urinary excretion, or rapid transcellular shifts. Severe hypophosphatemia is a medical emergency if it results from the true depletion of body stores, however, in cases of transmembrane distribution, it is asymptomatic [4]. Poor clinical outcomes due to ataxia, depressed myocardial contractility, diaphragmatic weakness is documented with phosphate levels <2 mg/dL; rhabdomyolysis usually occur at levels <1 mg/dL [5].

Spontaneous correction of the serum phosphate levels in our patient suggests that transmembrane redistribution is the most likely mechanism [6]. Insulin secretion (re-feeding), catecholamine excess, and respiratory alkalosis can cause hypophosphatemia through the same mechanism, however, none of these conditions were present in our patient.

Cadman et al. reported six independent cases very similar to ours, with symptomatic hypophosphatemia on presentation and rapid recovery [7]. As in our case, all these patients presented with cannabis hyperemesis syndrome [8]. Their preadmission (baseline) phosphate levels were normal, suggesting a relatively acute decrease in blood levels. The transient and self-correcting nature of the hypophosphatemia in patients reported by Cadman et al. suggests that ion redistribution likely played a role. The likely postulated mechanism was respiratory alkalosis leading to this redistribution of phosphate. However, as in our patient, none of these patients had an arterial blood gas evaluation [9-10].

Cannabis is the most commonly used illicit drug in the United States. As more states legalize marijuana, physicians are more likely to encounter patients with cannabis-related morbidity. Hypophosphatemia is a presenting feature of cannabinoid hyperemesis syndrome in some patients as mentioned in our case report (Poster. Ahmed T, Muhammad S, Safdar A, Shaukat A. The Mystery of the Low Phosphate: Marijuana is the Smoking Gun. ACP Internal Medicine Meeting; April 23, 2020).

## Conclusions

With the growing use of cannabis and its derivatives, physicians can expect to encounter more patients experiencing the full spectrum of its side-effects. With the cessation of marijuana use, the hypophosphatemia corrects fairly rapidly. Various studies suggest arrhythmogenic properties of cannabis, which makes severe electrolyte imbalance such as hypophosphatemia an important detail to consider. Without the cessation of cannabis use, the effects of severe prolonged hypophosphatemia are not known and hence both users and providers must be aware of this rare but potentially fatal adverse effect.
